# Phenotypic Identification of Spinal Cord-Infiltrating CD4^+^ T Lymphocytes in a Murine Model of Neuropathic Pain

**DOI:** 10.4172/2167-0846.S3-003

**Published:** 2014-02-19

**Authors:** KS Draleau, S Maddula, A Slaiby, N Nutile-McMenemy, JA De Leo, L Cao

**Affiliations:** 1 Department of Biomedical Sciences, College of Osteopathic Medicine, University of New England, Biddeford, ME, 04005, USA; 2 Department of Anesthesiology, Dartmouth Hitchcock Medical Center, Lebanon, NH 03756, USA; 3 Vice President of Academic Affairs, Professor of Biology, Emmanuel College, 400 The Fenway, Boston, MA 02215, USA

**Keywords:** CD4^+^ T lymphocytes, CD154 knockout mice, Type 1 helper T cells, Astrocytes

## Abstract

**Background:**

Neuropathic pain is one of the most devastating kinds of chronic pain. Neuroinflammation has been shown to contribute to the development of neuropathic pain. We have previously demonstrated that lumbar spinal cord-infiltrating CD4^+^ T lymphocytes contribute to the maintenance of mechanical hypersensitivity in spinal nerve L5 transection (L5Tx), a murine model of neuropathic pain. Here, we further examined the phenotype of the CD4^+^ T lymphocytes involved in the maintenance of neuropathic pain-like behavior via intracellular flow cytometric analysis and explored potential interactions between infiltrating CD4^+^ T lymphocytes and spinal cord glial cells.

**Results:**

We consistently observed significantly higher numbers of T-Bet^+^, IFN-γ^+^, TNF-α^+^, and GM-CSF^+^, but not GATA3^+^ or IL-4^+^, lumbar spinal cord-infiltrating CD4^+^ T lymphocytes in the L5Tx group compared to the sham group at day 7 post-L5Tx. This suggests that the infiltrating CD4^+^ T lymphocytes expressed a pro-inflammatory type 1 phenotype (Th1). Despite the observation of CD4^+^ CD40 ligand (CD154)^+^ T lymphocytes in the lumbar spinal cord post-L5Tx, CD154 knockout (KO) mice did not display significant changes in L5Tx-induced mechanical hypersensitivity, indicating that T lymphocyte-microglial interaction through the CD154-CD40 pathway is not necessary for L5Tx-induced hypersensitivity. In addition, spinal cord astrocytic activation, represented by glial fibillary acidic protein (GFAP) expression, was significantly lower in CD4 KO mice compared to wild type (WT) mice at day 14 post-L5Tx, suggesting the involvement of astrocytes in the pronociceptive effects mediated by infiltrating CD4^+^ T lymphocytes.

**Conclusions:**

In all, these data indicate that the maintenance of L5Tx-induced neuropathic pain is mostly mediated by Th1 cells in a CD154-independent manner via a mechanism that could involve multiple Th1 cytokines and astrocytic activation.

## Introduction

As one of the most devastating kinds of chronic pain, neuropathic pain, defined as pain caused by a lesion or disease of the somatosensory system [1], is a great challenge to physicians [2]. Decades of investigation using pre-clinical models has provided ample evidence of the contribution of the adaptive immune system (lymphocyte-mediated immune responses), to the development of neuropathic pain [3]. Specifically, using a well-established murine model, spinal nerve L5 transection (L5Tx), our laboratory has demonstrated that spinal cord-infiltrating CD4^+^ T lymphocytes contribute to the maintenance of L5Tx-induced mechanical hypersensitivity [4], indicating the involvement of helper CD4^+^ T lymphocyte-mediated immune responses in neuropathic pain. It is well known that helper CD4^+^ T lymphocytes can be classified into several functional subtypes, including type 1 (Th1, mediating primarily cell-mediated immunity), type 2 (Th2, mediating primarily humoral responses), and type 17 (Th17, mediating primarily inflammatory responses) helper T cells [5]. Each subtype of helper T cells has a distinct transcription factor and cytokine profile that drives the downstream responses they mediate. Previously, a limited number of studies that used indirect approaches suggested that Th1 is the dominant helper T cell subtype in mediating the development of neuropathic pain. First, Moalem et al. demonstrated that the passive transfer of *in vitro* maintained Th1, but not Th2, cells promoted nerve injury-induced behavioral hypersensitivity [6]. Others have subsequently shown the close association between increased spinal cord interferon IFN-γ (the signature cytokine produced by Th1 cells) and behavioral hypersensitivity, as well as an association between increased interleukin (IL)-4 (the signature cytokine produced by Th2 cells) expression and a reduction in nerve injury-induced sensory hypersensitivity [7,8]. More recently, the involvement of IL-17 (the signature cytokine produced by Th17 cells) in the development of peripheral nerve injury-induced neuropathic pain was described, suggesting a role of Th17 in neuropathic pain [9–11]. However, there have been no studies that directly examined the phenotype(s) of the infiltrating CD4^+^ T lymphocytes following peripheral nerve injury, which may in part be due to the technical difficulty of isolating the small number of lumbar spinal cord-infiltrating T cells. Thus, in the current study, we directly evaluated spinal cord-infiltrating CD4^+^ T lymphocytes based on their intracellular expression profiles of subtype-specific transcription factors and cytokines via flow cytometric analysis using the L5Tx model of neuropathic pain. As we did not detect significant changes in IL-17 expression in the lumbar spinal cord post-L5Tx in preliminary studies, we focused our investigation on the Th1 and Th2 subtypes.

Further, the underlying mechanism through which selected infiltrating helper T cell subtypes contribute to peripheral nerve injury-induced sensory hypersensitivity is still unclear. It has been proposed that infiltrating T lymphocytes interact with central nervous system (CNS) resident glial cells, including both astrocytes and microglia, to promote CNS pro-inflammatory responses that further contribute to central sensitization and persistent pain behaviors [3,12]. It is well-known that Th1 cells further activate macrophages through several co-stimulatory pathways. Previously, we have reported that microglial CD40 plays a critical role in the development of L5Tx-induced mechanical hypersensitivity [13]. As the ligation between CD40 expressed by macrophages and CD40 ligand (CD154) expressed by Th1 cells plays a key role in enhancing macrophage function in the peripheral immune system and microglia are the monocyte/macrophage lineage cells in the CNS, it is possible that infiltrating T lymphocytes play their pro-nociceptive role by interacting with microglia through the CD40–CD154 pathway. In fact, this very interaction has been linked to the pathogenesis of various CNS diseases, including multiple sclerosis and Alzheimer’s disease [14–19]. Thus, in this current study, we investigated whether CD154^+^CD4^+^ T lymphocytes contribute to the maintenance of long-term behavioral hypersensitivity with CD154 knockout (KO) mice. In addition, to examine whether lumbar spinal cord-infiltrating CD4^+^ T lymphocytes contribute to the maintenance of L5Tx-induced mechanical hypersensitivity through the regulation of spinal cord astrocytic activity, we also examined lumbar spinal cord astrocytic glial fibillary acidic protein (GFAP) immunoreactivity in time course studies in both wild type (WT) and CD4 KO mice.

## Materials and Methods

### Animals

WT male and female BALB/c mice were purchased from National Cancer Institute (NCI, Frederick, MD) and were allowed to habituate to the institutional animal facility for at least 1 week before experimental use (8–9 wks old). Breeding pairs for BALB/c CD154 KO mice were obtained from Dr. Abul Abbas at the University of California San Francisco and bred in the animal facility of University of New England (UNE). BALB/c CD4 KO mice were originally obtained from Dr. William T. Lee of the Wadsworth Center, New York State Department of Health and are currently maintained at the UNE animal facility by breeding homozygous KO mice. All mice were group-housed with food and water *ad libitum* and maintained on a 12-h light/dark cycle. Since no significant gender differences in all parameters measured were detected, both male and female mice (roughly 1:1 ratio) were used in all experiments. The Institutional Animal Care and Use Committee (IACUC) at UNE approved all experimental procedures. For the experiment conducted in the Dartmouth College (Figure 4A), The Institutional Animal Care and Use Committee (IACUC) at Dartmouth College approved the experimental procedures.

### L5Tx and assessment of mechanical sensitivity

WT BALB/c and BALB/c CD154 KO mice were randomly assigned into sham and L5Tx groups. L5Tx and sham surgeries were performed as previously described [4]. The mechanical sensitivity of each mouse was measured with von Frey filaments (Stoelting, Wood Dale, IL) using the Up-Down paradigm (detailed in [20]) as previously described [4]. The fifty percent threshold force needed for paw withdrawal was calculated and used to represent the mechanical sensitivity. No significant differences in basal mechanical sensitivity were observed between WT and CD154 KO mice. The experimenter performing the behavioral tests was blinded to the experimental groups (including both the genotype and type of surgery).

### Flow cytometry

As described previously [4], following transcardiac perfusion with phosphate buffered saline (PBS), mononuclear cells for flow cytometric analysis were prepared from an individual spleen, pooled lumbar lymph nodes (LNs) (5 mice), or pooled mouse lumbar spinal cord tissue (10 mice) by discontinuous Percoll (Amersham, Piscataway, NJ) gradients, and the total mononuclear cell number was determined using a hemacytometer with trypan blue (Sigma, St Louis, MO) prior to intracellular flow cytometric analysis. Due to the limited number of infiltrating cells available for analysis following the collection procedure (even with 10 mice pooled together) and the fact that the vast majority of infiltrating CD4^+^ T lymphocytes were observed in the ipsilateral side of dorsal horn area [4], lumbar spinal cord tissues were not separated as ipsilateral vs. contralateral in these experiments. In general, the intracellular staining was performed as follows. Briefly, following the final PBS wash, mononuclear cells were resuspended in staining buffer (2% fetal bovine serum (FBS)/0.09% NaN_3_/PBS) containing anti-mouse-CD16/CD32 monoclonal antibody (mAb) (clone 2.4G2; 1 µg/50µl/tube) and incubated on ice for 30 minutes. A combination of three fluorescent mAbs (Table 1) was added to each tube for surface staining (30 minutes on ice). After two PBS washes, the cells were fixed with 1% formaldehyde/PBS at room temperature for 20 minutes, washed with PBS again, and then permeabilized with a permeabilization buffer (0.5% saponin (Sigma)/2% FBS/0.09% NaN_3_/PBS) at room temperature for 10 minutes. Cells were then blocked with 10% isotype-matched serum (either mouse or rat serum) (in 0.5% saponin/PBS) on ice for 30 minutes. One selected fluorescent mAb (Table 1) was added to each tube for intracellular staining (30 minutes on ice). After being washed with permealization buffer, cells were resuspended in 1% formaldehyde/PBS and stored at 4°C until flow cytometric analysis. All samples were analyzed with an Accuri C6 flow cytometer (BD Biosciences-Accuri, Ann Arbor, MI) and FlowJo analysis software (Tree Star, Inc., Ashland, OR). For spleen and LN samples, a total of 20,000 events were collected and analyzed. For spinal cord mononuclear cells, due to the limited numbers of cells obtained, all events in each sample were analyzed. Non-stained cells, proper isotype controls, and surface-stained only controls were included as controls. For intracellular cytokine staining IFN-γ, IL-4, tumor necrosis factor (TNF)-α, and granulocyte-macrophage colony-stimulating factor (GM-CSF)), 0.1% brefeldin A (Sigma)/PBS was used in place of PBS from tissue harvesting up to the fixation step. All experiments were repeated several times in order to collect sufficient data for statistical analyses (i.e., when pooled samples were used, each pooled sample was considered as n=1).

### Determination of tissue levels of IFN-γ

Spleens and lumbar spinal cords were collected following transcardiac perfusion and processed for the assessment of IFN-γ levels via enzyme-linked immunosorbent assay (ELISA). Tissue homogenates were prepared as described previously [13] and stored at −80°C until analysis. Levels of IFN-γ were determined with a Duo Set ELISA kit (R&D Systems, Minneapolis, MN) following the manufacturers’ protocol. The IFN-γ level of each sample was normalized based on the protein concentration of the particular sample as determined by the BCA protein assay (Pierce-Thermo Scientific, Rockford, IL).

### Immunohistochemistry (IHC)

Fluorescent IHC for CD4, CD154, and GFAP was performed on the L5 segment of lumbar spinal cord sections following a previously published procedure [13]. For CD4, a FITC-rat-anti-mouse CD4 mAb (clone RM4–5; 1:100) (BD Biosciences, San Diego, CA), was used; for CD154, a primary mAb, purified Armenian hamster-anti-CD154 (clone MR1; 1:100) (BD Biosciences), and a secondary antibody (Ab), TRITC-goat-anti-Armenian hamster IgG (1:5000) (Jackson Immuno Research Laboratories, West Grove, PA), were used. For GFAP, a primary Ab, polyclonal rabbit-anti-GFAP (1:10,000) (DAKO UK Ltd., UK) and a secondary Ab, Cy3-goat-anti-rabbit IgG (1:800) (Jackson Immuno Research Laboratories), were used. DAPI-containing mounting media (either VECTASHIELD^®^ (Vector laboratories, Burlingame, CA) or Fluoromount-GT (VWR, Bristol, CT)) was used. “Non-stained” and “no primary Abs” controls were included when performing IHC, and no significant fluorescent signal was detected from these controls. For CD4 and CD 154 IHC, images were taken with an Olympus fluorescence microscope (U-ULH) (Olympus Optical Co.) and an Olympus Q-FIRE camera. For GFAP IHC, slices were examined with a Nikon Eclipse E800 fluorescence microscope (Nikon Instruments Inc., Melville, NY) and a SPOT RT Slider CCD microscope digital camera (Burlingame, CA). To determine GFAP expression in the spinal cord dorsal horn, black and white images were analyzed using Image J (NIH). Both the total dorsal horn area and GFAP immunostaining intensity were measured for each L5 dorsal horn image (represented images shown in Figure 5A). A ratio of total intensity/total dorsal horn area was then calculated and used as the GFAP immunoreactivity for each L5 dorsal horn slice. The average GFAP immunoreactivity of three slices from the same tissue sample were calculated and used as the GFAP expression for that particular sample in the final data analysis. The experimenter performing the analyses was blinded to the experimental groups.

### Statistical analyses

All data were graphed with SigmaPlot 10.0 (Systat Software, Inc. San Jose, CA) and analyzed with SigmaStat 3.5 (Systat Software, Inc. San Jose, CA). Appropriate analyses of variance (ANOVA) were performed followed by the Student-Newman-Keuls (SNK) *Post hoc* test. All data are presented as mean ± SEM when applicable. *p*<0.05 was considered as statistically significant.

## Results

### Flow cytometric analysis of CD4+ T lymphocytes in peripheral lymphoid tissues and the lumbar spinal cord

Previously, we have shown that spinal cord CD4^+^ T lymphocytes contribute to L5Tx-induced maintenance of mechanical hypersensitivity [4]. To identify the phenotype of CD4^+^ T lymphocytes involved in the development of L5Tx-induced mechanical hypersensitivity, we examined CD4^+^ T lymphocytes in both peripheral lymphoid tissues and the lumbar spinal cord via flow cytometry with antibodies that identify the Th1 and Th2 subtypes. WT BALB/c mice were randomly assigned to naive (no surgery), L5Tx, and sham groups. Since 7 days post-surgery is the peak time of detecting spinal cord-infiltrating CD4^+^ T lymphocytes, samples were collected at day 7 post-surgery.

**Figure 1:**
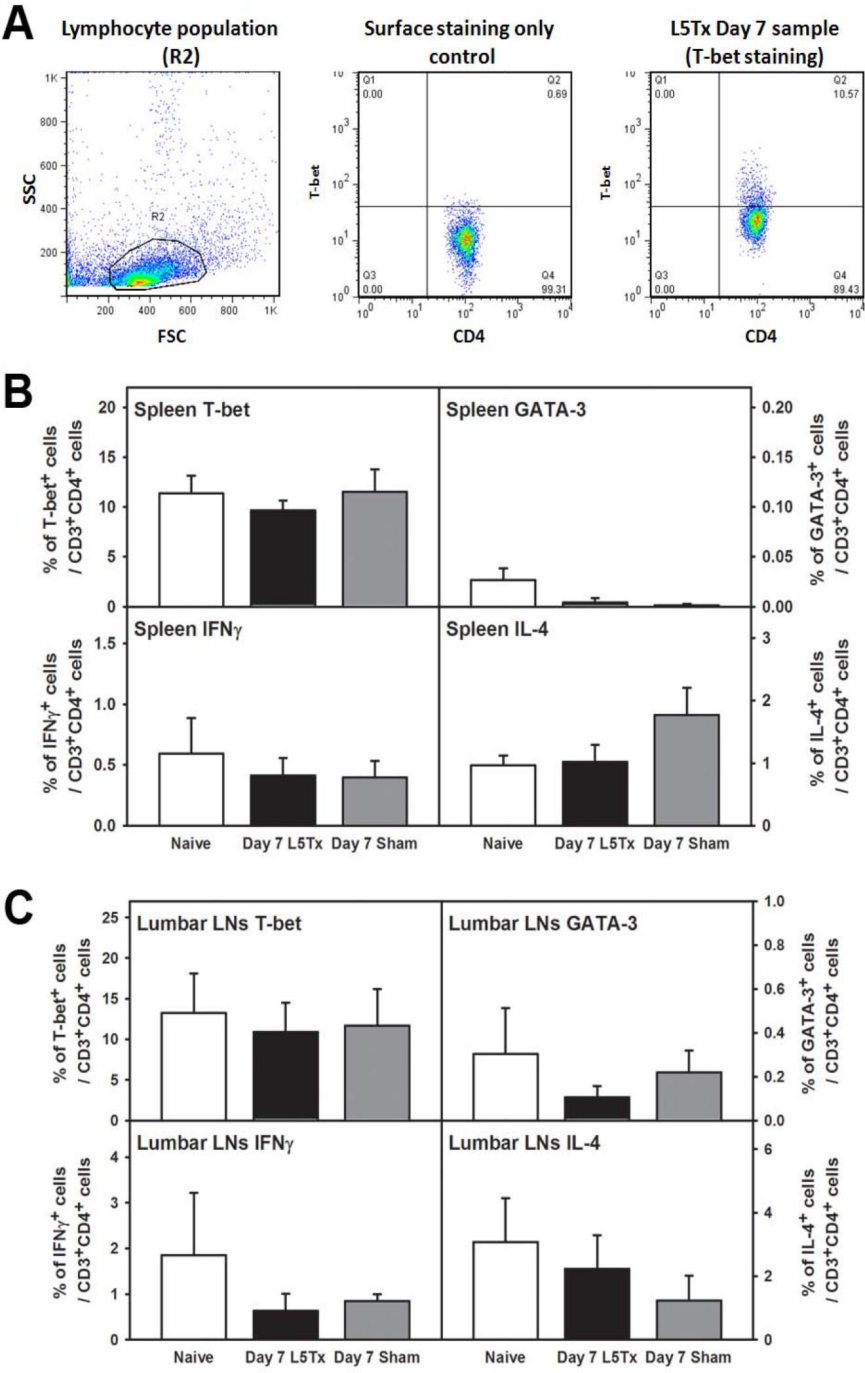
Flow cytometric analysis of CD4^+^ T lymphocytes in peripheral lymphoid tissuesafter L5Tx. WT BALB/c mice were subjected to either L5Tx or sham surgery. Splenocytes (from individual mice) and lumbar LN cells (pooled from 5 mice) were collected at 7 days after surgery and analyzed via intracellular flow cytometry with a combination of mAbs against CD45, CD3, CD4 and either T-bet, GATA-3, IFNγ, or IL-4 (see Methods). In A, representative images from a day 7 L5Tx spleen sample are shown to illustrate the analysis process. Total lymphocytes were first gated (R2 in the left panel), and CD4^+^ T lymphocytes (CD45^+^CD3^+^CD4^+^ population) were then selected for the analysis of each specific intracellular marker (middle and right panels, with T-bet used as an example): The final % of T-bet^+^ CD4^+^ T lymphocytes in one particular sample is the difference between the % of PE^+^(T-bet^+^) CD4^+^ T lymphocytes population obtained from T-bet-stained (CD45, CD3, CD4 and T-bet) sample (right panel) and the % of PE^+^ CD4^+^ T lymphocytes population obtained from the corresponding surface-stained only (CD45, CD3 and CD4) control (middle panel). In B and C, the final calculated % of T-bet^+^, GATA-3^+^, IFNγ^+^, or IL-4^+^ CD4^+^ lymphocytes within the total splenic lymphocytes (B, n = 7–10 mice per group) and the total lymphocytes from lumbar LNs (C, n = 3–4 repeats per group) are shown. All data are presented as mean ± SEM.

To examine CD4^+^ T lymphocytes in the periphery, spleens and lumbar LNs were collected and processed for intracellular flow cytometric analysis. CD4^+^ T lymphocytes were first identified (the CD45^+^CD3^+^CD4^+^ population), and then the expression of T-bet (transcription factor found in Th1, and not Th2, cells), GATA-3 (transcription factor found in Th2, and not Th1, cells), IFN-γ (one of the signature cytokines produced by Th1 cells), and IL-4 (one of the signature cytokines produced by Th2 cells) were examined within the CD4^+^ T lymphocytes (Figure 1A). Consistent with our previous findings regarding peripheral responses post-L5Tx [4], no significant differences in the percentages of T-bet^+^, GATA-3^+^, IFN-γ^+^, or IL-4^+^ CD4^+^ T lymphocytes were detected in either spleen or lumbar LNs when the naive, L5Tx and sham groups were compared (Figure 1B and 1C, one-way ANOVA, *p*>0.05 for all data sets within each graph). Since signs of splenic CD4^+^ T lymphocyte activation were previously observed at day 3 post-surgery [4], similar experiments were performed to examine splenic CD4^+^ T lymphocytes at day 3 post-surgery. However, no significant differences were detected in any of the above populations assessed (data not shown). Thus, the data indicate a lack of a significant Th1- or Th2-dominant response in the periphery after either L5Tx or sham surgery.

**Figure 2:**
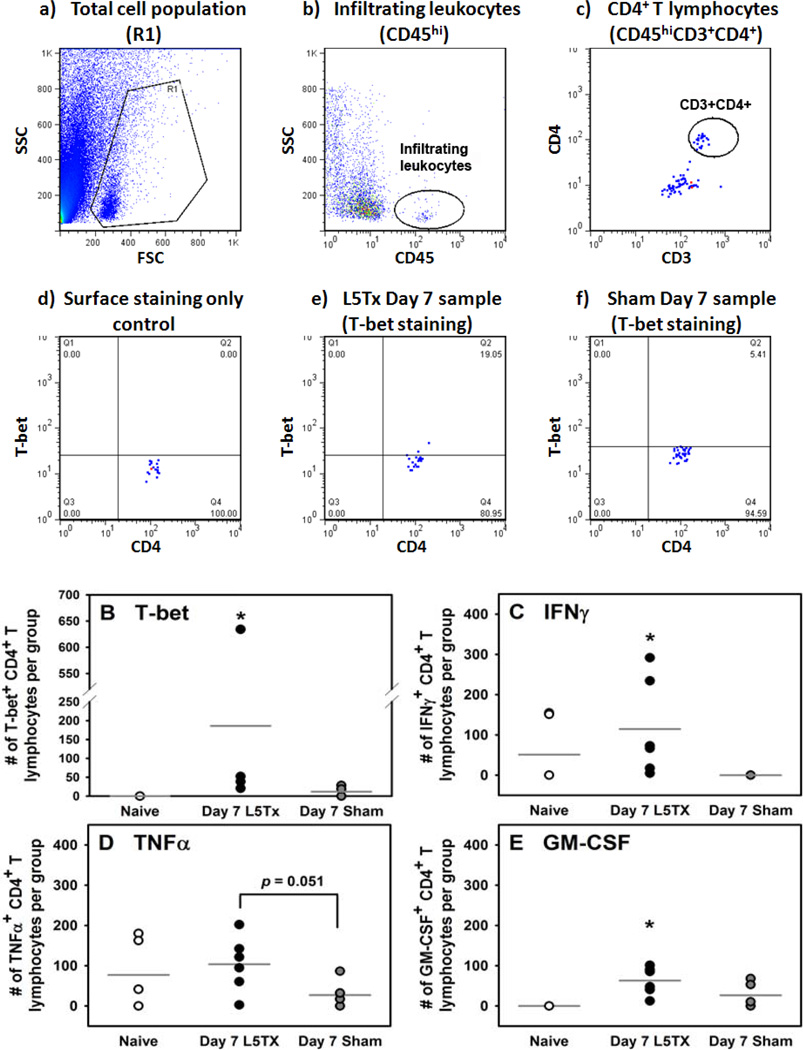
Flow cytometric analysis of CD4^+^ T lymphocytes in the lumbar spinal cord post-L5Tx. WT BALB/c mice were subjected to either L5Tx or sham surgery. Lumbar spinal cord mononuclear cells (pooled from 10 mice) were collected at 7 days after surgery and analyzed via intracellular flow cytometry with a combination of mAbs against CD45, CD3, CD4, and one of the following: T-bet, GATA-3, IFNγ, IL-4, TNFα, GM-CSF (see Methods). Experiments were repeated multiple times for statistical analyses. In A, representative images from lumbar spinal cord samples are shown to illustrate the analysis process. Total cell population was first gated (R1 in (a)), and then infiltrating leukocytes (CD45^hi^) were identified (b). CD4^+^ T lymphocytes (CD45^+^CD3^+^CD4^+^ population) (c) were further selected for the analysis of each specific intracellular marker ((d)–(f), T-bet is used as the example): The final % of T-bet^+^ CD4^+^ T lymphocytes in each sample is calculated as described in Figure 1A using “surface stained only” as control (d). Representative plots from a “Day 7 L5Tx” sample and a “Day 7 Sham” sample are shown in (e) and (f) respectively. Based on the total number of mononuclear cells collected, the number of T-bet^+^, GATA-3^+^, IFNγ^+^, IL-4^+^, TNFα^+^, and GM-CSF^+^ CD4^+^ T lymphocytes were calculated. In B–E, the calculated numbers of T-bet^+^ (B, n = 4), IFNγ^+^ (C, n = 5), TNFα^+^ (D, n = 6), and GM-CSF^+^ (E, n = 6) CD4^+^ lymphocytes per group of pooled lumbar spinal cords (10 mice) are shown. All data are presented as mean ± SEM. Each data point represents one pooled sample used. Note that, in some graphs, data points with similar values overlap. * indicates *p* < 0.05 between the indicated group and all other groups in the same graph.

We then examined the phenotype of the lumbar spinal cord-infiltrating CD4^+^ T lymphocytes in the naive, L5Tx, and sham groups. At day 7 post-surgery, lumbar spinal cord mononuclear cells were harvested and analyzed as described above via intracellular flow cytometric analysis (Figure 2). First, we examined the expression of the transcription factors T-bet and GATA-3 within the lumbar spinal cord-infiltrating CD4^+^ T lymphocytes. Within each set of experiments, the number of T-bet^+^CD4^+^ T lymphocytes in the L5Tx group was consistently higher than that of the naive and sham groups. As a result, there was a significant increase in the number of T-bet^+^CD4^+^ T lymphocytes in the L5Tx group compared to both the naive and sham groups. A slight, but not significant, increase in T-bet^+^CD4^+^ T lymphocytes was also observed in sham animals compared to naive mice (Figure 2B, one-way ANOVA, *p*<0.05). On the other hand, GATA-3^+^ CD4^+^ T lymphocytes were below the level of detection. Thus, these data indicated that the L5Tx-induced lumbar spinal cord infiltrating CD4^+^ T lymphocytes were predominantly Th1 cells. We further examined the cytokine expression of the infiltrating CD4^+^ T lymphocytes by measuring the number of IFN-γ^+^, IL-4^+^, TNF-α^+^, and GM-CSF^+^ CD4^+^ T lymphocytes. TNF-α and GM-CSF are two other cytokines predominantly associated with Th1 rather than Th2 cells. There were L5Tx-induced significant increases in the number of IFN-γ^+^ and GM-CSF^+^ lumbar spinal cord infiltrating CD4^+^ T lymphocytes (Figure 2C and E, one-way ANOVA, *p*<0.05). L5Tx induced similar, but not significant, changes in the number of TNF-α^+^ CD4^+^ T lymphocytes (Figure 2D, one-way ANOVA, *p*>0.05; *p*=0.051, L5Tx vs. sham), while no significant changes in the number of IL-4^+^ CD4^+^ T lymphocytes were detected (data not shown). These data further support lumbar spinal cord-infiltrating Th1 CD4^+^ T lymphocytes mediating L5Txinduced maintenance of mechanical hypersensitivity, and also suggest that this mediation might involve multiple Th1 cytokines.

### Lumbar spinal cord IFN-γ production in both WT and CD4 KO mice post-L5Tx

**Figure 3:**
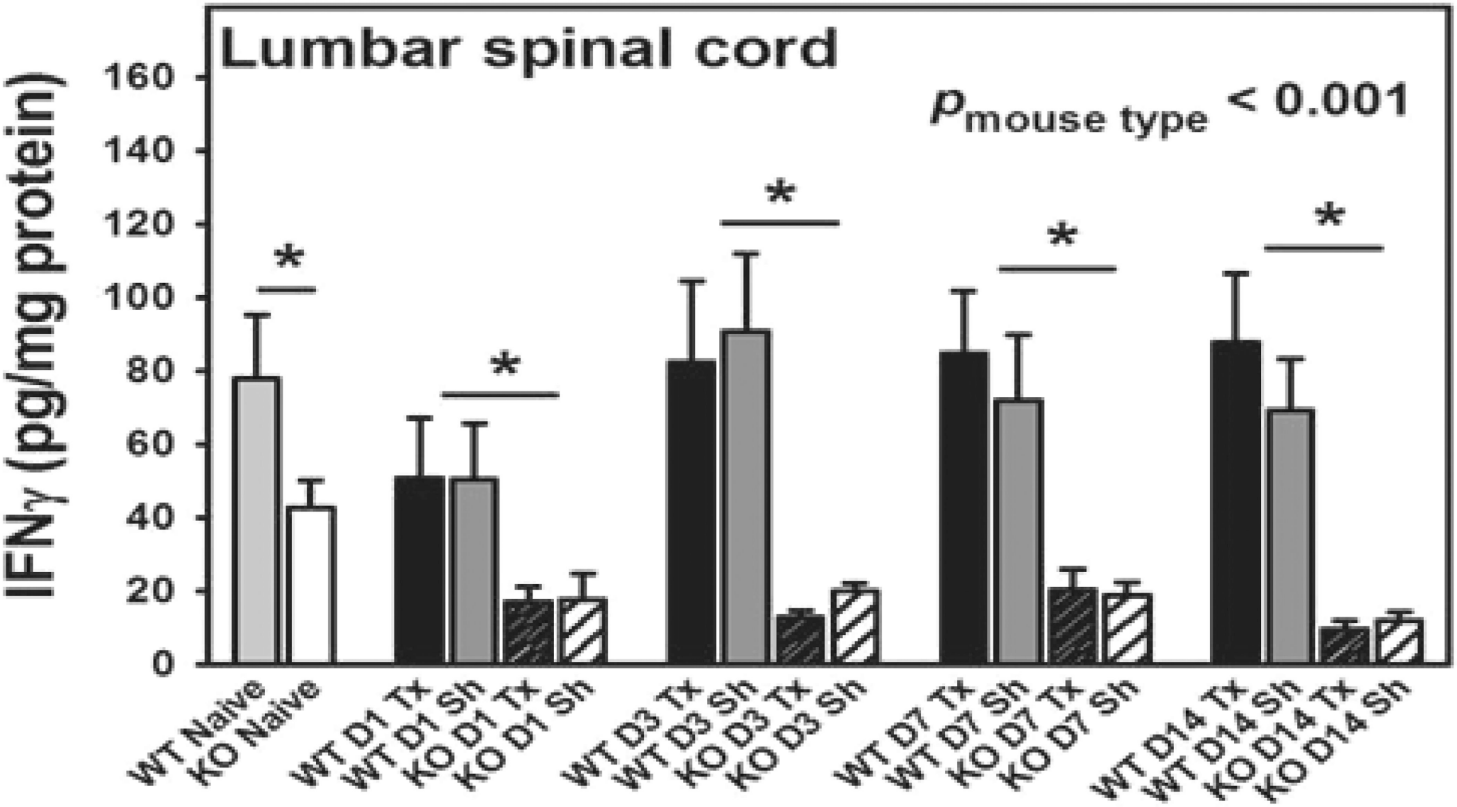
Lumbar spinal cord IFNγ production in both WT and CD4 KO mice post-L5Tx.WT BALB/c and BALB/c CD4 KO mice were subjected to either L5Tx or sham surgery. Lumbar spinal cords were collected at selected times post-surgery and then processed for IFNγ ELISA. Changes in lumbar spinal cord levels of IFNγ in WT (n = 9–15) and CD4 KO mice (n = 4–16) are shown here. All data are presented as mean ± SEM. No significant differences between L5Tx and sham treatments were detected within either WT or CD4 KO mice via one-way ANOVA. Two-way ANOVA with “time” and “genotype” as factors was further performed with all data (regardless of surgery). * indicates *p* < 0.05 between WT and CD4 KO mice at the indicated time point (regardless of surgery). “D” = Day, “WT” = WT mice, “KO” = CD4 KO mice, Tx = L5Tx, and Sh = sham.

We further measured the total levels of IFN-γ in the lumbar spinal cord at selected times (day 0 (naive), 3, 7, and 14) after L5Tx or sham surgery in both WT BALB/c and BALB/c CD4 KO mice via ELISA. First, no significant differences between the L5Tx and sham treatments were detected within either of the genotypes (Figure 3, one-way ANOVA for WT mice, *p*=0.685, and for CD4 KO mice, *p*=0.601). Therefore, further statistical analyses were performed on all data using “time” and “genotype” as factors regardless of treatment. It was found that CD4 KO mice expressed significantly lower levels of IFN-γ in the lumbar spinal cord compared to WT mice at all-time points, while there were no significant changes in the total levels of IFN-γ detected within either genotype over time, (Figure 3, two-way ANOVA, *p_genotype_*<0.001, *p_time_*=0.146, *p_genotype × time_*=0.243). In addition, IFN-γ was not detectable in the splenic tissue of any of the mice. These data support that a reduction in IFN-γ levels (which can be associated with a blunted Th1 response) may contribute to the observed reduced mechanical hypersensitivity in CD4 KO mice compared to WT mice following L5Tx [4].

### Mechanical sensitivity of CD154 KO mice

**Figure 4:**
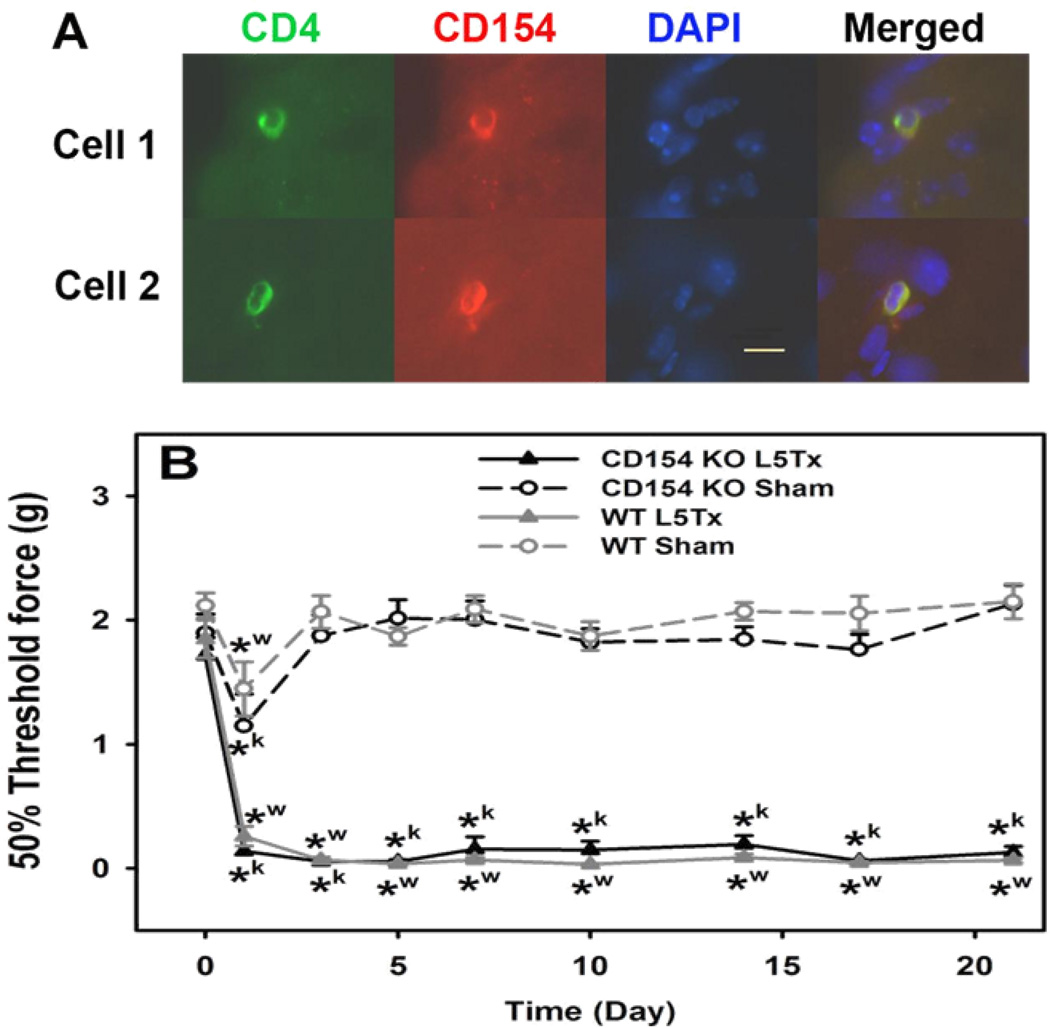
Detection of CD154^+^CD4^+^ T lymphocytes in the lumbar spinal cord and the mechanical sensitivity of CD154 KO mice post-L5Tx. In A, lumbar spinal cord sections from WT BALB/c mice 7 days post-L5Tx were processed for immunohistochemical analysis of CD4 (in green), CD40L (CD154, TRITC in red), and nuclei (DAPI in blue). CD154^+^CD4^+^ T lymphocytes (dual-labeled with CD4 and CD154, in yellow when merged) were detected within the parenchyma of the dorsal horn region in the spinal cord 7 days post-L5Tx. Examples of two detected cells (from ipsilateral side) are shown here. Merged images are shown on the far right (40×, Scale bar = 10 µm). In B, WT BALB/c mice and BALB/c CD154 KO mice were subjected to either L5Tx or sham surgery. All mice were tested for their mechanical sensitivity with von Frey filaments using the Up-Down method as described in the Methods. Data are presented as mean ± SEM (n = 8). *^w^ indicates *p* < 0.05 between the indicated group and the corresponding day 0 group within the WT mice. *^k^ indicates *p* < 0.05 between the indicated group and the corresponding day 0 group within the CD154 KO mice.

In the periphery, Th1 cells can promote macrophage proinflammatory responses through the ligation of CD154 expressed by Th1 cells and CD40 expressed by macrophages [21]. Previously, we have reported the critical role of microglial CD40 in the development of L5Tx-induced mechanical hypersensitivity [13]. To further identify the infiltrating CD4^+^ T lymphocyte-mediated downstream responses that contribute to the development of sustained mechanical hypersensitivity following L5Tx, we investigated the potential involvement of the interaction between microglial CD40 and CD154 expressed by spinal cord-infiltrating CD4^+^ T lymphocytes. First, we confirmed that L5Txinduced spinal cord infiltrating CD4^+^ T lymphocytes express CD154 via IHC. Serial sections of L5 spinal cord segments from two WT BALB/c mice 7 days post-L5Tx were stained for CD4 and CD154. Consistent with what we have shown previously, the majority of CD4^+^ T lymphocytes were observed in the ipsilateral side of the dorsal horn [4]. CD154^+^CD4^+^ T lymphocytes were detected in the ipsilateral side of the dorsal horn region (see representative images in Figure 4A). Due to the limited numbers of infiltrating CD4^+^ T lymphocytes, we could not accurately quantify the CD154^+^CD4^+^ T lymphocytes via IHC. To further investigate the CD40–CD154 pathway, an *in vivo* study with CD154 KO mice was conducted. We tested the mechanical sensitivity of BALB/c CD154 KO mice and WT BALB/c mice following either L5Tx or sham surgery with von Frey filaments. No basal differences in mechanical sensitivity were observed between the CD154 KO mice and WT mice. Interestingly, L5Tx induced significant mechanical hypersensitivity starting at day 1 and up to 21 days post-surgery in both CD154 KO and WT mice compared to the corresponding sham groups, while no significant differences in mechanical sensitivity between CD154 KO and WT mice were detected (Figure 4B; Two-way repeated ANOVA, *p*_time_<0.001, *p*_group_<0.001, and *p*_time × group_<0.001). Thus, it is unlikely that Th1 CD4^+^ T lymphocytes mediate L5Tx-induced behavioral hypersensitivity via the CD40–CD154 pathway.

### Lumbar spinal cord GFAP expression in WT and CD4 KO mice post-L5Tx

**Figure 5:**
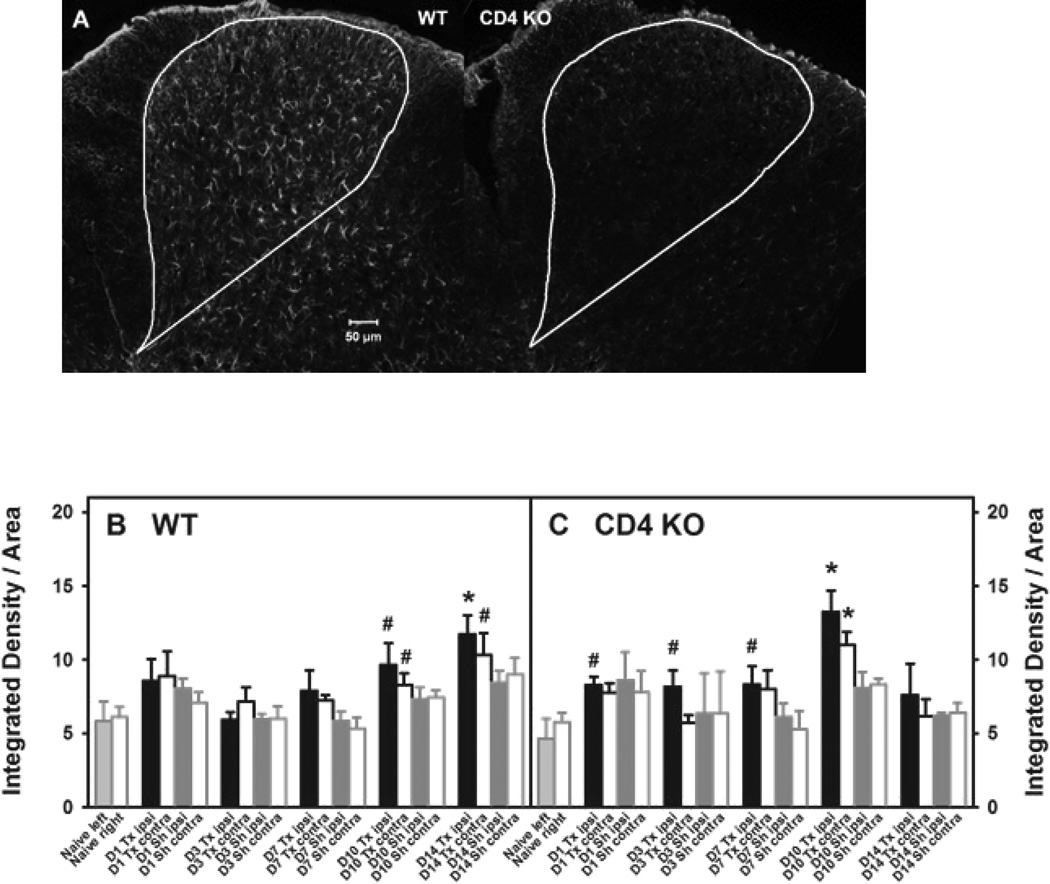
Lumbar spinal cord GFAP expression in WT and CD4 KO mice post-L5Tx. WT BALB/c and BALB/c CD4 KO mice were subjected to either L5Tx or sham surgery. The L5 segment of the lumbar spinal cord was processed for GFAP IHC. Representative ipsilateral L5 sections from WT and CD4 KO mice 14 days following L5Tx are shown in A (10×, scale bar = 50µM). The total dorsal horn area used for analysis is outlined in each image. Quantitative data for GFAP expression in WT and CD4 KO mice are presented in B and C, respectively (mean ± SEM, n = 4–7 per group). * indicates *p* < 0.05 between the indicated group and the corresponding naive group; # indicates 0.05 < *p* < 0.10 between the indicated group and the corresponding naive group. For naïve mice, left = ipsilateral side and right = contralateral side. “D” = Day, Tx = L5Tx, Sh = sham, ipsi = ipsilateral side and contra = contralateral side.

To explore whether lumbar spinal cord-infiltrating CD4^+^ T lymphocytes can contribute to the maintenance of L5Tx-induced mechanical hypersensitivity by regulating astrocytic responses, we evaluated L5 lumbar spinal cord GFAP expression (increase of GFAP expression is an indicator of astrocytic activation) at selected times (day 0 (naive), 1, 3, 7, 10, and 14) after either L5Tx or sham surgery in both WT and BALB/c CD4 KO mice via IHC (Figure 5). The basal levels of GFAP expression were similar between WT and CD4 KO mice. In WT mice, L5Tx induced significant increases in GFAP expression in both the ipsilateral and contralateral sides (no significant differences between sides) over time (Figure 5B; Two-way ANOVA, *p*_time_<0.001, *p*_group_=0.049, and *p*_time × group_<0.945). Consistent with what has been previously reported [22]; this increase is most significant after day 7 post-L5Tx. However, in CD4 KO mice, although L5Tx induced an increase in GFAP expression similar to WT mice up to day 10 post-L5Tx, the increase in GFAP expression was not observed in CD4 KO mice at day 14 post-L5Tx (Figure 5C; Two-way ANOVA, *p*_time_<0.001, *p*_group_=0.084, and *p*_time × group_<0.757). These data suggest a potential link between spinal cord-infiltrating CD4^+^ T lymphocytes and L5Tx-induced astrocytic responses, particularly those that occur during the later maintenance phase of neuropathic pain.

## Discussion

The involvement of adaptive immunity, particularly spinal cord-infiltrating CD4^+^ T lymphocytes, in the development of nerve injury-induced behavioral hypersensitivity has been well-accepted [3,4]. Several pre-clinical studies have provided evidence, such as the passive transfer of individual T lymphocyte subtypes and the measurement of tissue levels of Th1 cell-producing cytokines [6,8], that suggests that the Th1 subtype of CD4^+^ T lymphocytes plays a role in peripheral nerve injury-induced sensory hypersensitivity. However, there has been no direct examination of peripheral nerve injury-induced infiltrating CD4^+^ T lymphocytes. In the current study, we analyzed L5Tx-induced lumbar spinal cord-infiltrating CD4^+^ T lymphocytes by intracellular flow cytometric analysis via an array of Th1 and Th2 CD4^+^ T lymphocyte cellular markers. Our results directly show an increase in Th1, but not Th2, CD4^+^ T lymphocytes in the lumbar spinal cord following L5Tx and suggest that Th1 CD4^+^ T lymphocytes mediate their pronociceptive effects via multiple cytokines (such as IFN-γ, TNF-α, and GM-CSF). Interestingly, although all of the Th1 cytokines measured have been associated with nerve injury-induced pain behaviors [8,23–25], some of them have also been associated with nerve regeneration and the recovery of nerve function following nerve injury [24–26]. Further investigation of such cytokine-mediated effects will help elucidate the specific role of infiltrating CD4^+^ T lymphocytes in the development of neuropathic pain, and such knowledge could help in the design of therapies that would target the cytokines/mechanisms responsible for the maintenance of neuropathic pain while avoiding eliminating factors that have neuroprotective effects.

Unlike what was previously reported [8], we did not detect a significant increase in IFN-γ expression in the lumbar spinal cord post-L5Tx. This may be due to the different animal species used (rats vs. mice), the different assays used (polymerase chain reaction vs. ELISA), and/or the existence of multiple cellular sources of IFN-γ (including infiltrating T lymphocytes and macrophages and resident microglia and astrocytes). Nevertheless, we did detect significantly lower levels of IFN-γ in CD4 KO mice compared to WT mice at all times evaluated (including baseline), which could in part explain our previous observation that CD4 KO mice displayed significantly reduced hypersensitivity post-L5Tx. However, the cytokine mediators through which CD4^+^ T lymphocytes specifically promote the pathogenesis of the maintenance phase but not the initiation phase of neuropathic pain still need further investigation. We suspect that multiple cytokines are likely to be involved in this mechanism and that the cytokines are likely to work in a synergistic manner.

To further identify potential CD4^+^ T lymphocyte-mediated downstream pathways, we examined the role of T lymphocyte-expressing CD154. CD40 is a cell surface receptor expressed by activated microglia [27–30]. CD40 ligand (CD154) is a surface protein primarily expressed by activated CD4^+^ T lymphocytes [21]. Although it is known that the CD154-CD40 interaction between Th1 cells and macrophages promotes macrophage responses [21] and that the interaction between microglial CD40 and CNS infiltrating-T lymphocyte CD154 has been linked to the pathogenesis of various CNS diseases [14–19], our data indicate that it is unlikely that lumbar spinal cord-infiltrating CD4^+^ T lymphocytes mediate their pro-nociceptive effects through CD154-mediated interactions with spinal cord microglial CD40. Similar to our observation, it has been reported that CD40, but not CD154, was required for mounting an optimal response against *M tuberculosis* infection, and this discrepancy was attributed to an interaction between CD40 and a non-CD154 CD40 ligand [31]. The discrepancy in the development of behavioral hypersensitivity between CD40 KO and CD154 KO mice suggest that such an alternative CD40 ligand may be involved in CD40-mediated pro-nociceptive effects. In addition, we previously did observe a reduction in the L5Tx-induced increase of microglia in the lumbar spinal cord in CD4 KO mice [32]. Thus, further studies are necessary to explore other factors involved in the interaction between CD4^+^ Th1 lymphocytes and microglia.

It is well-known that glial cells (including both astrocytes and microglia) play critical roles in the development of neuropathic pain [33–35]. Here, to further identify CD4^+^ T lymphocyte-mediated pathways, we also examined the lumbar spinal cord astrocytic response in CD4 KO mice via the analysis of GFAP expression. Our data suggest that the lack of CD4^+^ T lymphocytes is associated with a shorter-lasting L5Tx-induced increase of GFAP expression in the lumbar spinal cord. So far, no study has reported a potential interaction between infiltrating CD4^+^ T lymphocytes and astrocytes in the development of neuropathic pain. We suspect that Th1 CD4^+^ T lymphocytes could regulate astrocytes by both direct and indirect mechanisms. For instance, it is known that nitric oxide (NO) can induce GFAP expression [36] and that the Th1-related cytokines IFN-γ and TNF-α are known to be involved in NO production in the CNS [37]. Further, our previous observation of a reduction in the L5Tx-induced increase of microglia in the lumbar spinal cord in CD4 KO mice coupled with our GFAP results presented here suggest that CD4^+^ T lymphocytes may regulate astrocytic responses via microglia-mediated mechanisms [32]. Comprehensive *in vitro* and *in vivo* studies could be used to establish the interactions between Th1 CD4^+^ T lymphocytes and astrocytes in the future.

In summary, our data support that lumbar spinal cord infiltrating Th1, and not Th2, CD4^+^ T lymphocytes mediate the maintenance of peripheral nerve injury-induced neuropathic pain. These infiltrating Th1 CD4^+^ T lymphocytes are likely to mediate their effects in mechanisms involving multiple Th1 cytokines and glial activation. Further investigation of CD4^+^ T lymphocyte-mediated pathways may result in novel, more efficacious treatments for neuropathic pain.

## Figures and Tables

**Table 1:** Primary monoclonal antibodies (mAb) used for flow cytometry.

	Primary mAb	Source	Clone	Isotype	Stock concentration	Working dilution
**Surface staining**^a^	APC-anti-mouse CD45	eBioscience	30-F11	Rat IgG2b	0.2 mg/ml	1:50
	FITC-anti-mouse CD4	BD Biosciences	GK1.5	Rat IgG2b	0.5 mg/ml	1:50
	PerCP-anti-mouse CD3	BD Biosciences	145-2C11	Armenian Hamster IgG1	0.2 mg/ml	1:50
**Intracellular staining**^b^	PE-anti-mouse T-bet	eBioscience	eBio4B10	Mouse IgG1	0.2 mg/ml	1:100
	PE-anti-mouse GATA-3	eBioscience	TWAJ	Rat IgG2b	3 μg/ml	1:100
	PE-anti-mouse IFN-γ	eBioscience	XMG1.2	Rat IgG1	0.2 mg/ml	1:400
	PE-anti-mouse IL-4	eBioscience	11B11	Rat IgG1	0.2 mg/ml	1:100
	PE-anti-mouse TNF-α	eBioscience	MP6-XT22	Rat IgG1	0.2 mg/ml	1:100
	PE-anti-mouse GM-CSF	eBioscience	MP1-22E9	Rat IgG2a	0.2 mg/ml	1:200

a For each sample tube, all three mAbs for surface staining were added at the same time.

b For each sample tube, one of these mAbs was used during intracellular staining.
